# Clinical application of multi-detector computed tomography coronary angiography and global longitudinal strain for diagnosis of multi-vessel coronary artery spasm: case reports

**DOI:** 10.1093/ehjcr/ytaf401

**Published:** 2025-08-19

**Authors:** Sangyong Jo, Kyung Hee Lim, Moo Hyun Kim, Eun-Ju Kang, Jinwoo Jeong

**Affiliations:** Department of Cardiology, Dong-A University College of Medicine, 32, Daesingongwon-ro, Seo-gu, Busan 49201, Korea; Department of Cardiology, Dong-A University College of Medicine, 32, Daesingongwon-ro, Seo-gu, Busan 49201, Korea; Department of Cardiology, Dong-A University College of Medicine, 32, Daesingongwon-ro, Seo-gu, Busan 49201, Korea; Department of Radiology, Dong-A University College of Medicine, 32, Daesingongwon-ro, Seo-gu, Busan 49201, Korea; Department of Emergency Medicine, Dong-A University College of Medicine, 32, Daesingongwon-ro, Seo-gu, Busan 49201, Korea

**Keywords:** Global longitudinal strain, Multivessel coronary artery spasm, Multi-detector computed tomography coronary angiography, Case report

## Abstract

**Background:**

Diagnosis of epicardial coronary artery spasm, which is considered the cause of vasospastic angina, can be achieved through the coronary angiography (CAG) with a pharmacologic provocation test. However, this procedure is thought to have a potential risk of complications. Although multi-detector computed tomography coronary angiography (MDCTA) is proposed as the alternative test for the diagnosis of spastic coronary artery, myocardial ischaemia does not always coincide with coronary artery stenosis. MDCTA combined with echocardiographic left ventricular global longitudinal strain (LV GLS) is an innovative non-invasive diagnostic method expected to detect multivessel coronary artery spasm in patients with vasospastic angina.

**Case summary:**

We report two cases of vasospastic angina caused by multivessel coronary artery spasm, which were confirmed using MDCTA and echocardiographic LV GLS.

**Discussion:**

In patients with suspected vasospastic angina, MDCTA and echocardiographic LV GLS during spontaneous attacks could be used to document the coronary artery vasospasm and associated myocardial ischaemia. These non-invasive diagnostic techniques could be considered a new alternative for patients with suspected vasospastic angina who exhibit certain characteristics, such as nitrate-responsive and resting chest pain and no history of significant coronary atherosclerosis. This approach may be particularly useful when these patients present to the emergency department during a spontaneous vasospasm.

Learning points

- Echocardiographic LV GLS in combination with nitrate-free MDCTA enhances non-invasive detection of coronary artery spasm and myocardial ischaemia in patients with vasospastic angina, potentially reducing invasive testing.
- A combined approach using MDCTA and LV GLS should be considered in patients with vasospastic angina with nitrate-responsive, resting chest pain, particularly those presenting to the emergency department with a spontaneous spasm attack and no prior history of significant coronary atherosclerosis.
- Injection of nitroglycerin successfully recovered the decreased LV GLS associated with multivessel coronary spasm.
- Echocardiography with LV GLS after nitrate administration could be used to prevent complications associated with repetitive post-nitrate MDCTA and demonstrate the reversibility of myocardial ischaemia after coronary artery vasodilation.

## Introduction

Vasospastic angina is characterized as nitrate-responsive angina with transient ischaemic electrocardiographic changes in the ST segments. Epicardial coronary artery spasm, which is presumed to cause vasospastic angina, can be demonstrated through the coronary angiography (CAG) with a pharmacologic provocation test.^1^ However, the CAG with provocation test in the acute phase carries a potential risk of procedure-related complications such as hypotension, cardiogenic shock, arrhythmia, myocardial infarction, and even death.^2^ If transient ischaemic electrocardiogram (ECG) changes are documented as the result of a spontaneous coronary artery spasm accompanied by nitrate-responsive angina, the CAG with provocation test has no benefit during the acute phase.^3^ However, documenting transient ischaemic ECG changes during spontaneous episodes of coronary artery spasm is rare, and a second diagnostic method is often required. Multi-detector computed tomography coronary angiography (MDCTA) has been proposed as an alternative non-invasive method for diagnosing coronary artery spasm.^4^ However, controversy remains regarding the diagnostic performance of MDCTA for detection of vasospastic angina. Above all, significant myocardial ischaemia does not always coincide with coronary artery stenosis. Left ventricular global longitudinal strain (LV GLS) is an echocardiographic method used to detect subclinical ventricular systolic dysfunction caused by myocardial ischaemia.^5^ Therefore, we report two cases in which an innovative complementary technique involving MDCTA and echocardiographic LV GLS was used to detect multivessel coronary artery spasms in patients with vasospastic angina.

## Summary figure

**Figure:**
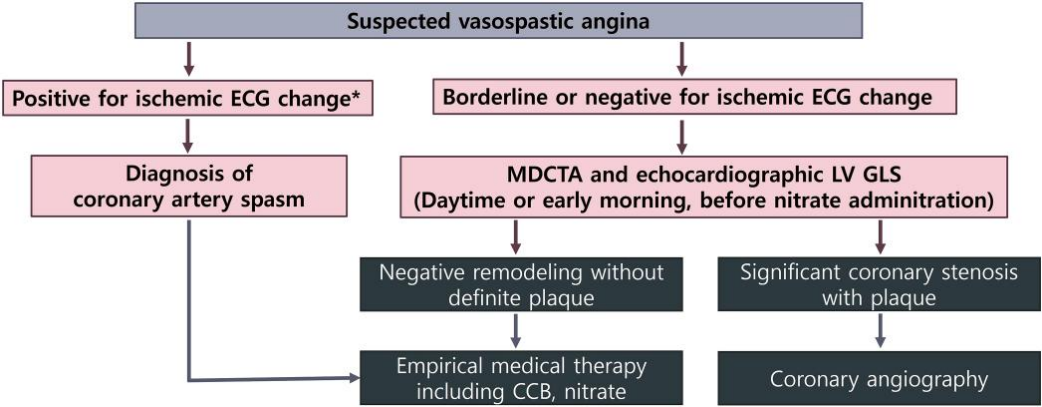
Diagnostic algorithm of vasospastic angina. *Ischaemic ECG change: transient ST elevation of 0.1 mV or more, ST depression of 0.1 mV or more, or new appearance of negative U waves, recorded in at least two contiguous leads on 12-lead ECG. CCB, calcium channel blocker; ECG, electrocardiogram; LV GLS, left ventricular global longitudinal strain; MDCTA, multi-detector computed tomography coronary angiography.

## Case presentation

### Case 1

**Figure 1:**
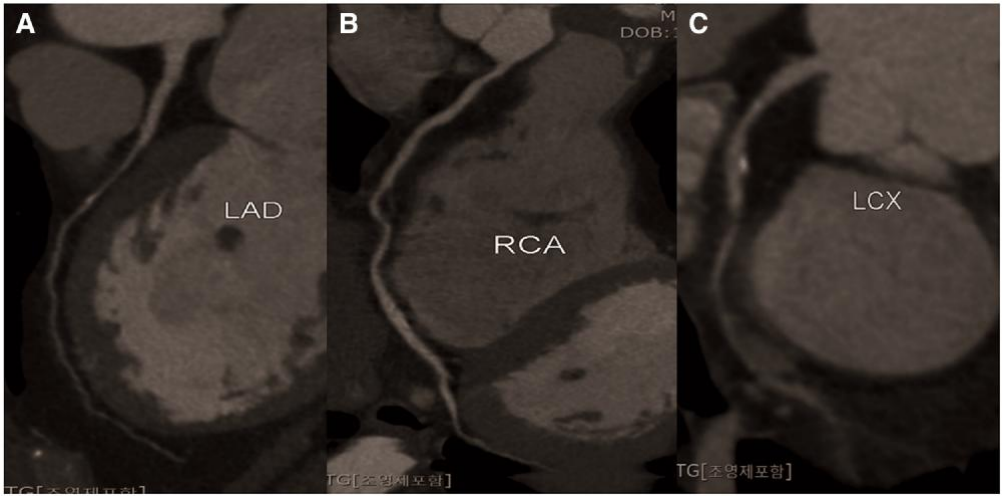
Case 1: curved multiplanar reformation imaging from multi-detector computed tomographic coronary angiography. Baseline computed tomographic imaging reveals diffuse luminal narrowing in multivessel coronary arteries. (*A*) Left anterior descending artery. (*B*) Right coronary artery. (*C*) Left circumflex artery.

**Figure 2:**
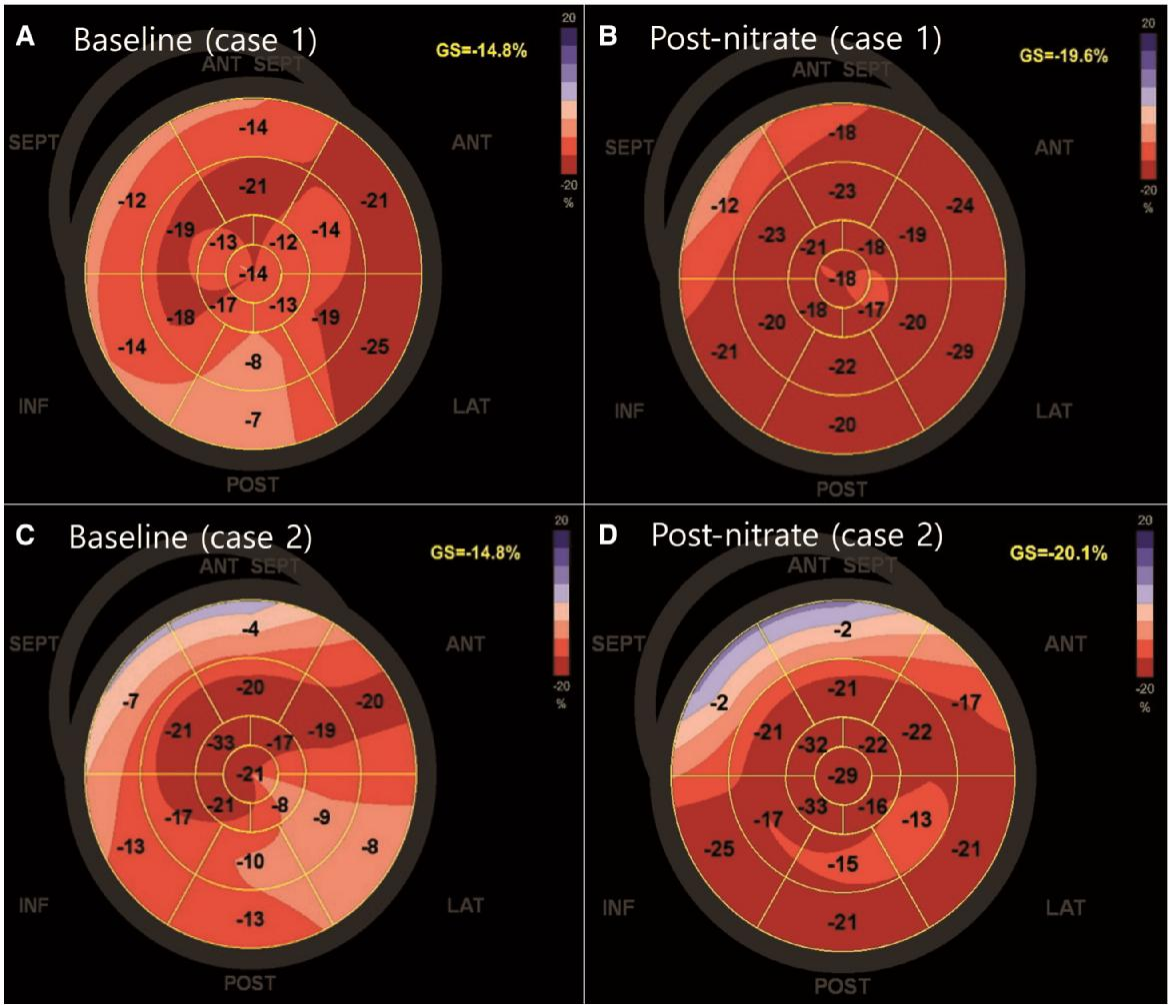
Comparison of LV GLS imaging. Baseline LV GLS calculated from three standard apical views was slightly decreased (<15%). After nitrate administration, LV GLS improved compared to baseline LV GLS in both cases. (*A*) Case 1: baseline LV GLS. (*B*) Case 1: post-nitrate administration. (*C*) Case 2: baseline LV GLS. (*D*) Case 2: post-nitrate administration. LV GLS, left ventricular global longitudinal strain.

**Figure 3:**
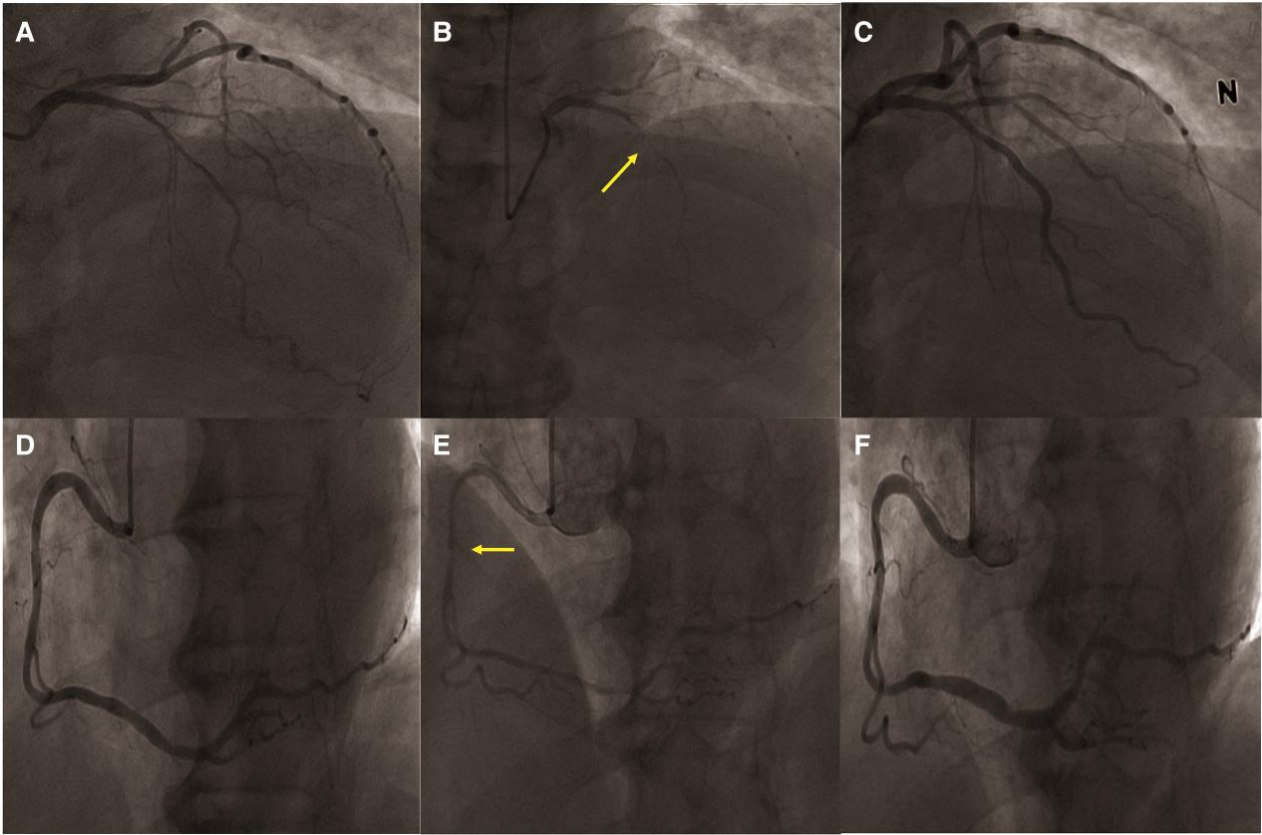
Case 1: coronary angiography with ergonovine provocation test. (*A*) Left coronary artery before ergonovine provocation test. (*B*) Left coronary artery during maximal epicardial spasm. (*C*) Left coronary artery after intracoronary nitroglycerine injection. (*D*) Right coronary artery before ergonovine provocation test. (*E*) Right coronary artery during maximal epicardial spasm. (*F*) Right coronary artery after intracoronary nitroglycerine injection.

A 59-year-old man with nitrate-responsive angina was referred to the emergency room. Although he had undergone CAG for chest pain about 7 years prior, the pharmacologic provocation test had not been performed. Previous CAG revealed no significant coronary artery disease. His angina symptoms had remained uncontrolled despite medical treatments such as angiotensin-converting enzyme inhibitors, antiplatelet medication, statins, diltiazem, nicorandil, and molsidomine. His vital signs, physical examination, and cardiac enzyme results did not reveal any abnormal findings. Initial electrocardiogram showed T wave flattening or inversion in the inferior leads (II, III, and aVF). We performed MDCTA during ongoing chest pain early in the morning on the first day of hospitalization. MDCTA imaging without administration of nitrate revealed diffuse luminal narrowing in the left anterior descending artery (LAD), mid-right coronary artery (RCA), and left circumflex artery (LCX), compatible with coronary artery spasm (*Figure 1*). Echocardiography and CAG with the ergonovine provocation test were performed on the next day after admission in the early morning as a research protocol. Although the MDCTA findings alone strongly suggested vasospastic angina, invasive CAG with provocation was performed for confirmation in this complex case. Also, vasodilators were not administered until the start of the CAG with provocation test to avoid their effects on the accuracy of the test. Echocardiography showed slightly decreased LV GLS even though the LV ejection fraction was normal (*Figure 2A*). Baseline CAG showed diffuse stenosis in the left coronary artery and a beaded appearance in the right coronary artery (*Figure 3A, 3D*). Finally, intracoronary administration of ergonovine induced a diffuse significant spasm with >90% luminal narrowing along the left and right coronary arteries, particularly in the mid-LAD lesions (*Figure 3B, 3E*). Because the diffuse coronary artery spasm was resolved after intracoronary nitroglycerine injection (*Figure 3C, 3F*), it was confirmed that the diffuse luminal narrowing observed on MDCTA was not a baseline anatomical variant. The patient was diagnosed with vasospastic angina. The echocardiographic LV GLS improved from −14.8% to −19.6% after nitroglycerine injection (*Figure 2B*). The patient was discharged with additional isosorbide mononitrate and a long-acting calcium channel blocker (amlodipine), and there were no further recurrences of angina.

### Case 2

**Figure 4:**
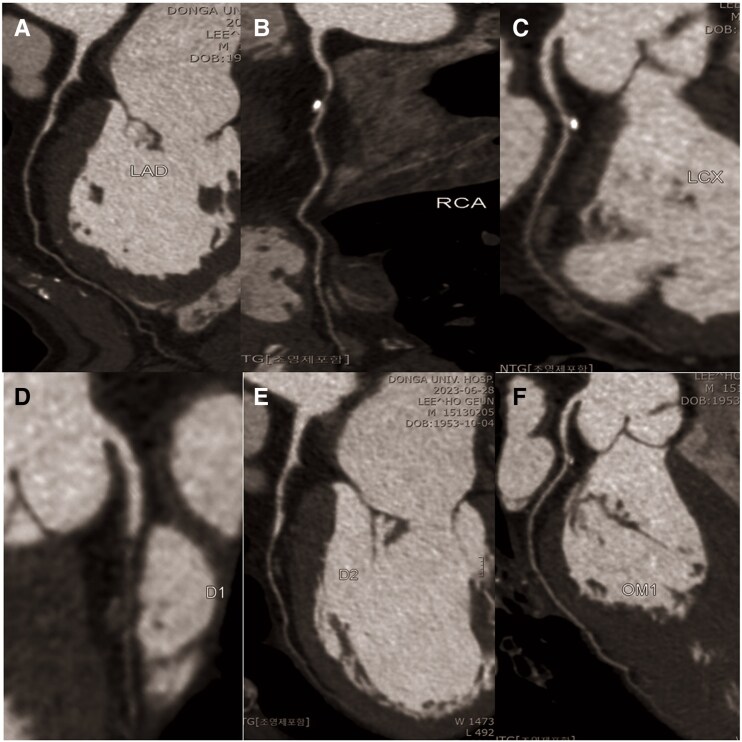
Case 2: curved multiplanar reformation imaging from multi-detector computed tomographic coronary angiography. (*A*) Left anterior descending artery. (*B*) Right coronary artery. (*C*) Left circumflex artery. (*D*) First diagonal branch. (*E*) Second diagonal branch. (*F*) Obtuse marginal branch.

**Figure 5:**
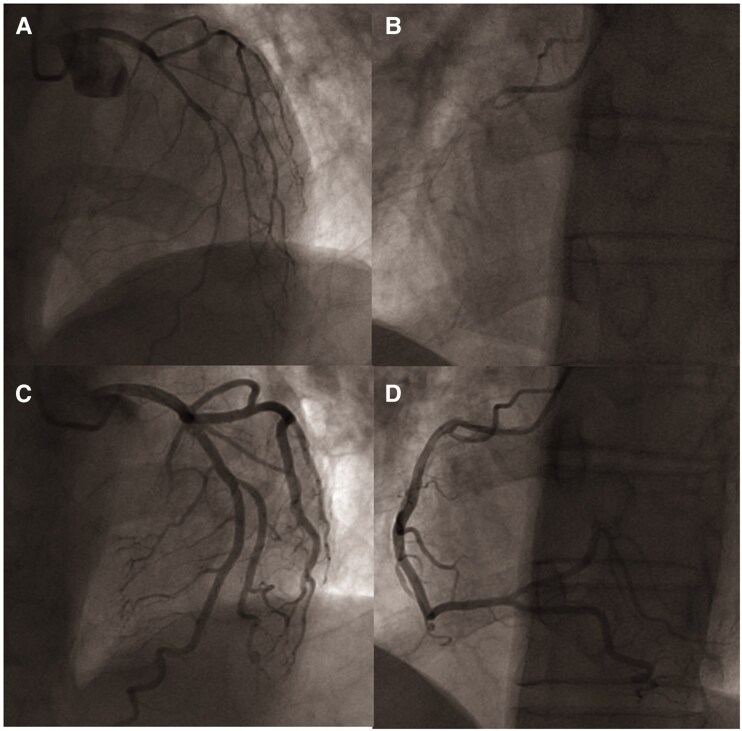
Case 2: coronary angiography with ergonovine provocation test. (*A*) Baseline left coronary artery angiography. (*B*) Coronary artery angiography after RCA-first ergonovine provocation test. (*C*) Left coronary artery after intracoronary nitroglycerine injection. (*D*) Right coronary artery after intracoronary nitroglycerine injection.

A 70-year-old male with a history of hypertension and angina visited the cardiology outpatient department with severe chest pain associated with alcohol consumption. Even though CAG without provocation test several years prior had shown no significant coronary atherosclerotic disease, he currently was experiencing frequent worsening chest pain despite treatment with high-dose diltiazem and isosorbide mononitrate. Sublingual nitroglycerin usually relieved the chest pain within several minutes. He was hospitalized after discontinuing his medical treatments for 5 days. A 12-lead ECG upon admission showed a sinus rhythm and incomplete right bundle branch block. His serial troponin levels during hospitalization were negative. MDCTA, echocardiography, and CAG with provocation test were all performed sequentially on the day after admission, in accordance with a research protocol. MDCTA and baseline echocardiography were performed without nitrate administration. The MDCTA showed multifocal luminal narrowing without definite plaque in the LAD, RCA, LCX, and diagonal and obtuse marginal arteries (*Figure 4*). Echocardiography revealed significantly decreased LV GLS, although the LV ejection fraction was within the normal range (62%) (*Figure 2C*). In the initial CAG, before the provocation test, diffuse narrow lesions were seen in multiple vessels (*Figure 5A*). Induced total occlusion developed in the proximal RCA after the first dose of ergonovine (10 µg) (*Figure 5B*). The vasospasm was unresponsive to intracoronary nitroglycerin administration, and the patient experienced chest pain and sinus bradycardia, with a heart rate of <40 b.p.m. After three additional injections of intracoronary nitroglycerine, the sinus rhythm was restored, and his symptoms resolved. The final CAG showed that his coronary flow had completely recovered (*Figure 5C, 5D*). His echocardiographic LV GLS improved from −14.8% to −20.1% after nitrate administration (*Figure 2D*). He was started on 2.5 mg of S-amlodipine daily instead of diltiazem to reduce coronary vasospasm. Alcohol abstinence was recommended for management of vasospastic angina. He did not experience recurrence of chest pain, and his blood pressure was controlled.

## Discussion

These case reports demonstrate the diagnostic value of MDCTA and echocardiographic LV GLS without nitrate administration in two patients with vasospastic angina confirmed by CAG with the ergonovine provocation test. Vasospastic angina is a major cause of ischaemia with non-obstructive coronary arteries, and it is also characterized as a hyperreactive response of the epicardial coronary artery segment to vasoconstrictive stimuli.^6^ According to a Japanese Circulation Society guideline, pharmacological coronary spasm provocation tests are recommended for patients with suspected vasospastic angina who have not been diagnosed with coronary spasm in a non-invasive evaluation.^3^ A typical ischaemic ECG change is sufficient to conclude myocardial ischaemia in a patient with definite vasospastic angina. However, there are some limitations to diagnosing ischaemic ECG changes in clinical practice. A previous study showed that 52.8% of patients who were diagnosed as positive on the ergonovine echocardiography test did not exhibit ischaemic ECG changes.^7^ Likewise, neither of the two patients in these case reports showed typical ischaemic ECG changes, according to the current guideline for vasospastic angina.^3^ Therefore, additional non-invasive diagnostic techniques are needed.

MDCTA has been investigated as a non-invasive diagnostic method for coronary artery vasospasm.^8^ The NAVIGATOR study showed that MDCTA can contribute to the non-invasive detection of vasospastic angina. Those researchers performed MDCTA before [baseline computed tomography (CT)] and after (IV nitrate CT) an intravenous infusion of nitrate and suggested the following positive criterion on the baseline CT: (i) negative remodelling and no clear evidence of plaque or (ii) a diffuse small diameter (<2 mm) of the main coronary artery with a beaded appearance. Vasospastic angina is defined as complete resolution of such positive findings on IV nitrate CT.^4,9^ MDCTA showed relatively good specificity and positive predictive value (PPV), but its sensitivity was lower than that of the coronary spasm provocation test.^3,4^ Additionally, repeated CT scans might increase complication risks from radiation and contrast. Non-invasive methods such as transthoracic echocardiography with GLS, cardiac magnetic resonance imaging, and nuclear medicine examinations are also used to evaluate myocardial ischaemia in patients with suspected vasospastic angina. Compared to other non-invasive modalities, MDCTA combined with echocardiographic GLS provides an easily accessible and widely available alternative for evaluating such patients. This combined approach addresses the limitations of diagnosing vasospastic angina using MDCTA alone, which has lower sensitivity in the absence of a functional assessment. Therefore, an integrated technique of MDCTA with echocardiographic LV GLS to enhance non-invasive detection was used in this case report for diagnosing vasospastic angina.

In this case series, MDCTA and baseline echocardiography with LV GLS were performed in the early morning (between 7 and 8 a.m.) on the same day. Clinical, laboratory, and non-invasive imaging data at admission are summarized in *Table 1*. To enhance diagnostic sensitivity, MDCTA without nitrate infusion was performed in a patient (Case 1) who visited the emergency room with an angina attack caused by spontaneous vasospasm. LV GLS was measured in both cases to prove silent myocardial ischaemia induced by coronary artery vasospasm.^10^ Moreover, follow-up echocardiography with LV GLS after nitrate administration was performed to prevent complications associated with repeated CT scans and demonstrate the reversibility of myocardial ischaemia after coronary artery vasodilation. The follow-up echocardiography after intravenous infusion of nitrate was performed within 24 h of CAG. Two-dimensional echocardiography with LV GLS was performed in a standard manner using commercially available echocardiographic devices, and all examinations were conducted by an experienced echocardiography specialist. Finally, CAG with an ergonovine provocation test was performed on the same day as or the following day after MDCTA, depending on the circumstances.

**Table 1:** Overview of the clinical, laboratory, and non-invasive imaging data of the two cases at admission

	First case	Second case
Systolic blood pressure (mmHg)	150	138
Diastolic blood pressure (mmHg)	80	78
Heart rate (b.p.m.)	72	62
High-sensitivity troponin I (ng/L; normal < 0.0342 ng/L)	0.0128	0.0021
NT-proBNP (pg/mL; normal <125 pg/mL)	41.8	113
Typical ischaemic ECG change^a^	No	No
Baseline LV GLS (%)	−14.8	−14.8
Follow-up LV GLS after nitrate administration (%)	−19.6	−20.1

^a^Ischaemic ECG change: transient ST elevation of 0.1 mV or more, ST depression of 0.1 mV or more, or new appearance of negative U waves, recorded in at least two contiguous leads on 12-lead ECG.

This case series highlights the potential utility of combining nitrate-free MDCTA with echocardiographic LV GLS as a non-invasive diagnostic strategy in patients with suspected vasospastic angina. These combined non-invasive diagnostic imaging tools could be considered a new alternative option for identifying vasospastic angina in patients who have certain characteristics, such as nitrate-responsive and resting chest pain and no history of significant coronary atherosclerosis. This approach may be particularly useful in situations known to precipitate coronary artery vasospasm (e.g. early morning or after alcohol consumption) or in patients presenting to the emergency department with spontaneous attacks. Notably, neither of the two patients in our case report had significant coronary atherosclerosis.

Our case report has limitations. Although our cases support the complementary role of MDCTA and LV GLS in diagnosing vasospastic angina as a non-invasive imaging technique, invasive CAG with provocation test was performed to provide confirmatory evidence in these complex cases. Additionally, echocardiographic strain measurement can be influenced by patient-specific factors such as age, sex, haemodynamic status, and concomitant cardiovascular disease. These factors may serve as confounding factors in the interpretation of the results. Further prospective studies are warranted to validate the diagnostic accuracy and clinical applicability of this combined MDCTA and LV GLS strategy and to determine whether they can ultimately serve as non-invasive alternatives to CAG with provocation testing in select patients.

## Supplementary Material

ytaf401_Supplementary_Data

## Data Availability

The processed data required to reproduce the above findings cannot be shared at this time as they are part of an ongoing study.

## Lead author biography

Sangyong Jo is a physician in the Department of Cardiology of Dong-A University Hospital, Busan, Korea. His areas of interest include ischaemic heart disease and structural heart disease.
